# Complete Genome Sequence of *Geobacter* sp. Strain SVR, an Antimonate-Reducing Bacterium Isolated from Antimony-Rich Mine Soil

**DOI:** 10.1128/MRA.00142-21

**Published:** 2021-04-08

**Authors:** Tomoro Warashina, Shigeki Yamamura, Haruo Suzuki, Seigo Amachi, Kazuharu Arakawa

**Affiliations:** a Institute for Advanced Biosciences, Keio University, Tsuruoka, Yamagata, Japan; b Systems Biology Program, Graduate School of Media and Governance, Keio University, Fujisawa, Japan; c Center for Regional Environmental Research, National Institute for Environmental Studies, Tsukuba, Ibaraki, Japan; d Faculty of Environment and Information Studies, Keio University, Fujisawa, Japan; e Graduate School of Horticulture, Chiba University, Matsudo, Chiba, Japan; f Exploratory Research Center on Life and Living Systems, National Institutes of Natural Sciences, Myodaiji, Okazaki, Japan; University of Delaware

## Abstract

We report here the complete genome sequence of *Geobacter* sp. strain SVR, isolated from antimony mine soil in Nakase Mine, Hyogo Prefecture, Japan. SVR strains proliferate using antimonate [Sb(V)] as an electron acceptor, providing insights into the antimony reduction mechanism.

## ANNOUNCEMENT

Antimony (Sb) is widely distributed in the environment due to natural as well as industrial causes. In the environment, antimony exists mainly in the inorganic forms Sb(V) and Sb(III), with Sb(III) being more cytotoxic to humans (1). Few microorganisms have been reported to proliferate using Sb(V) as an electron acceptor; therefore, the mechanisms of Sb(V) transport and reduction from Sb(V) to Sb(III) remain limited. We previously isolated an Sb(V)-reducing bacterium, *Geobacter* sp. strain SVR, from antimony mine soil in Nakase Mine, Hyogo Prefecture, Japan, to elucidate the molecular mechanisms of antimony metabolism (2). Nakase Mine has gold and antimony veins and has produced the largest amount of antimony in Japan.

Strain SVR was cultured for 3 days at a temperature of 30°C under anaerobic conditions (20 ml medium in a 60-ml glass vial, with 40 ml headspace filled with 20% N_2_ and 80% CO_2_). The composition of the medium used in this study was the same as that used in the previous study (3), except that it did not contain resazurin solution. High-molecular-weight DNA was extracted and purified according to the Genomic-tip 20/G manual (Qiagen) using approximately 1.14 × 10^9^ cells from the strain SVR culture medium. Sequencing libraries were prepared and multiplexed using the rapid barcoding kit (SQK-RAB004; Oxford Nanopore Technologies) and were sequenced using a FLO-MIN106 flow cell on a GridION X5 device (Oxford Nanopore Technologies). A total of 45,705 reads (*N*_50_ length of 31,866 bp) were obtained, and all reads were used for *de novo* assembly using Canu version 2.1.1 (4). The assembled single contig was manually circularized by eliminating an overlapping end. The assembly was further error corrected using Pilon version 1.23 (5) using an Illumina short-read sequence (BioProject accession number PRJDB5044) published in a previous study (3). The assembly quality was assessed using CheckM (marker lineage *Geobacteraceae*) (6), where the completeness score was 99.49%. The genome was annotated and rotated to start with the *dnaA* gene using the DDBJ Fast Annotation and Submission Tool (DFAST) (7). All software programs were used with default settings.

The complete genome sequence of strain SVR was obtained, consisting of a circular chromosome 4,717,360 bp long with a G+C content of 57.9%. DFAST annotation predicted 4,423 protein-coding sequences (CDSs), 6 rRNA genes, and 52 tRNA genes. Among the 4,423 protein-coding sequences, 1,791 CDSs were hypothetical proteins. Comparing the previously reported draft genome sequence of strain SVR with this assembly, this assembly is 55,144 bp longer and has 67 more genes than the assembly in the previous study (3). In order to compare the assemblies of this study with those of previous studies, the large-scale BLAST score ratio (LS-BSR) pipeline (8) was used. The CDS predicted from the assembly in this study was used as the query sequence to run tBLASTn (9). Fourteen genes (LOCUS_10770, LOCUS_11750, LOCUS_16390, LOCUS_27550, LOCUS_28590, LOCUS_28600, LOCUS_28610, LOCUS_29650, LOCUS_30580, LOCUS_32800, LOCUS_32810, LOCUS_34940, LOCUS_34960, LOCUS_34970) with a BSR value of less than 0.8 were identified by the LS-BSR pipeline, and these genes were considered to be newly identified by this assembly. Fourteen unique genes were annotated as a hypothetical protein (12 genes) and an AMP-binding protein (2 genes) by DFAST. Strain SVR is one of the few bacteria that proliferate using antimony [Sb(V)] as an electron acceptor, and the complete genome sequence presented in this study may provide insight into antimony metabolism.

### Data availability.

The chromosome sequence reported here was deposited in DDBJ under accession number AP024469, and the raw reads were deposited in the Sequence Read Archive (SRA) under BioProject accession number PRJNA698610.
